# Cardiac autonomic neuropathy and risk of cardiovascular disease and mortality in type 1 and type 2 diabetes: a meta-analysis

**DOI:** 10.1136/bmjdrc-2021-002480

**Published:** 2021-12-30

**Authors:** Mahin Chowdhury, Sarah Nevitt, Aikaterini Eleftheriadou, Prathap Kanagala, Hani Esa, Daniel J Cuthbertson, Abd Tahrani, Uazman Alam

**Affiliations:** 1 Department of Cardiovascular and Metabolic Medicine, University of Liverpool, Liverpool, UK; 2 Department of Health Data Science, University of Liverpool, Liverpool, UK; 3 Department of Medicine, University Hospital Aintree, Liverpool University NHS Foundation Trust, Liverpool, UK; 4 Centre of Endocrinology, Diabetes and Metabolism, University of Birmingham, Birmingham, UK; 5 Institute of Metabolism and Systems Research, University of Birmingham, Birmingham, UK; 6 Department of Diabetes and Endocrinology, University Hospitals Birmingham NHS Foundation Trust, Birmingham, UK; 7 Division of Diabetes, Endocrinology and Gastroenterology, Institute of Human Development, University of Manchester, Manchester, UK; 8 Department of Cardiovascular & Metabolic Medicine, Institute of Life Course and Medical Sciences and Pain Research Institute, University of Liverpool and Liverpool University Hospital NHS Foundation Trust, Liverpool, UK

**Keywords:** cardiac autonomic neuropathy, mortality, cardiovascular system, diabetes complications

## Abstract

We aimed to determine the prognostic association between cardiac autonomic neuropathy (CAN) and cardiovascular disease events (CVE) and mortality in type 1 and type 2 diabetes through a systematic review and meta-analysis. This systematic review and meta-analysis was registered with PROSPERO (CRD42020216305) and was conducted with Preferred Reporting Items for Systematic Reviews and Meta-Analyses (PRISMA) methodological criteria. CAN was defined on the basis of 1 (early/possible CAN) or ≥2 (definite CAN) positive autonomic function tests as per the Toronto Consensus guidelines. Studies included those with prospective CVE or mortality data. Methodological variables/risk of bias were assessed using ROBINS-I (Risk Of Bias In Non-randomized Studies - of Interventions) and RoB-2 (Risk-Of-Bias tool for randomized trials) appraisal tools. Electronic database searches yielded 18 467 articles; 84 articles were screened full-text, 26 articles fulfilled the inclusion criteria for quantitative synthesis. Sixteen studies from patients with (n=2875) and without (n=11 722) CAN demonstrated a pooled relative risk (RR) of 3.16 (95%CI 2.42 to 4.13; p<0.0001) of future CVE in favour of CAN. Nineteen studies provided all-cause mortality data from patients with (n=3679) and without (n=12 420) CAN, with a pooled RR of 3.17 (95%CI 2.11 to 4.78; p<0.0001) in favour of CAN. The risk of both future CVE and mortality was higher in type 1 compared with type 2 diabetes and with a definite CAN (vs possible CAN) diagnosis. Three studies were considered to have risk of serious bias. This study confirms the significant association between CAN and CVE and all-cause mortality. The implementation of population-based CAN screening will identify a subgroup with disproportionately higher cardiovascular and mortality risk that will allow for earlier targeted intervention.

## Introduction

Cardiovascular disease (CVD) is a major cause of global mortality with coronary artery disease accountable for the greatest burden, with a US prevalence in (type 1 and type 2) diabetes of between 30% and 51%, after adjustment for age.^1^ In the Framingham Study,^2^ cardiovascular disease events (CVE) were between two to three times higher in people with diabetes compared with those without diabetes. Data from the WHO Multinational Study of Vascular Disease in Diabetes demonstrated that CVD was the most prevalent cause of death in type 1 diabetes (44%) and in type 2 diabetes (52%).^3^ The excess CVD mortality risk is multifactorial secondary to obesity, hypertension and dyslipidemia, leading to the development of both atherosclerotic CVD and cardiac autonomic neuropathy (CAN).

CAN is a serious, although often overlooked complication of diabetes mellitus. The reported prevalence ranges from 2% to 91% in type 1 diabetes and 25% to 75% in type 2 diabetes^4 5^ dependent on clinical and demographic factors. It is considered to be one of the most common diabetic complications. CAN remains under-reported and the reasons for this are multifactorial and include but are not limited to a lack of an easy implementable population-based screening and the time needed to screen. The development of CAN is associated with dysfunction of innervation of the heart and vasculature, leading to impaired cardiovascular function. CAN is a progressive condition starting from a subclinical disease demonstrated by a reduction in heart rate variability (HRV) during deep breathing. Similar to somatic neuropathies, autonomic nerves are affected in a length-dependent manner. The vagus nerve (the longest parasympathetic nerve) is the first to be affected in CAN,^6^ leading to resting tachycardia, sympathetic nervous system predominance and abnormalities in left ventricular systolic and diastolic functions.^6 7^ Increase in sympathetic tone continues with advancing CAN, whereby denervation of the sympathetic nervous system also occurs.^8^ Clinical sequelae with progression to advanced CAN include orthostatic hypotension, exercise intolerance, chronic kidney disease (CKD), and silent myocardial ischemia (SMI), all of which contribute to significant morbidity and premature mortality. The reference standard diagnostic measures of CAN are cardiovascular autonomic reflex tests, consisting of five non-invasive tests (commonly known as Ewing’s battery) that assesses both the sympathetic and parasympathetic nervous system. Ewing and Clarke suggested using all five tests to diagnose CAN. Further in-depth analysis of CAN assessment is beyond the scope of this article and we would direct the readers to Vinik *et al* 2013.^9^

In a meta-analysis conducted in 2003, Maser *et al* synthesised the evidence base to assess the relationship between CAN and risk of mortality in diabetes.^10^ CAN was associated with future risk of mortality with both definite (≥2 autonomic function test (AFT) abnormality) and possible (1 AFT abnormality) CAN with a stronger association observed with definite CAN.^10^ We conducted this study with the primary aim to update this work published nearly two decades ago and to increase its scope to include the relationship between CAN and risk of CVE, while including newer studies reflecting improved standards of care. No other meta-analyses have been conducted in the interim. Therefore, we used robust CVE definitions as utilized in Cardiovascular Outcomes Trials (CVOT)^11^ to synthesise the most up-to-date evidence base to determine the prognostic association of CAN with CVE and mortality, through a systematic review and meta-analysis conducted to international standard Preferred Reporting Items for Systematic Reviews and Meta-Analyses (PRISMA) methodology.

## Methods

### Search strategy

This study was reported according to the international standard PRISMA guidelines. An *a priori* protocol was developed and registered with The International Prospective Register of Systematic Reviews (PROSPERO) (CRD42020216305). A comprehensive search strategy was developed with an information specialist at the University of Liverpool. An electronic database search was performed using the following: MEDLINE (access via OVID); PubMed; Scopus; Cochrane; and Cumulative Index to Nursing and Allied Health Literature (CINAHL). Searches were conducted by two authors independently (MC, AE) from inception of the database to November 2020 and were restricted to English language. Prespecified search terms relevant to CAN, CVE and mortality were used for each database (online supplemental ESM table 1). Free-text search terms were also implemented in each database to find additional relevant articles. All relevant results from each database were merged using EndNote to enable the removal of any duplicates. All additional studies were obtained after searching reference lists of relevant reviews/systematic reviews. Articles reporting CAN and CVE and/or CAN and all-cause mortality data were included.

10.1136/bmjdrc-2021-002480.supp1 Supplementary data

### Definition of CAN and comparators

In line with the Toronto Diabetic Neuropathy Expert Group, definitions of CAN were dichotomized into either: possible/early CAN (one positive AFT) and definite CAN (two or more positive AFTs).^12^ Data were extracted from comparator groups who were people with diabetes without CAN.

### Inclusion/exclusion criteria

An *a priori* inclusion/exclusion criteria were used on all relevant results to select final articles for full-text assessment. These are detailed below:

Inclusion criteria

1. Randomized controlled trials or prospective cohort studies displaying CVE or mortality data in people with and without CAN.
2. CAN being defined with AFT.
3. Conducted in adults (≥18 years) with diabetes.
4. Full-text publications.

Exclusion criteria

1. Not an original paper.
2. Non-human study.
3. Lack of available/extractable CAN and CVE and/or mortality data.
4. Non-English language publication.

Two authors (MC, AE) screened each title and abstract from the literature search to identify relevant articles. If any doubt arose regarding the eligibility of any given study, the article was included to critique the full text. The full-text articles were then assessed (by MC, AE) independently, using the inclusion/exclusion criteria. In the event of disagreement between the first two authors, the senior author (UA) was the final arbiter. UA confirmed the eligibility of all full-text articles prior to data extraction and quality assessment. The details of this process are highlighted in the PRISMA flow chart (online supplemental ESM figure 1).

### Data extraction and quality assessment

Data from all eligible articles were extracted into a standardised spreadsheet by two authors (MC, AE) independently. Studies’ first author, study name, type of study, year of publication, country and setting were also extracted. Clinical, demographic and metabolic data were extracted (online supplemental ESM tables 3-5). Subsequently, the methods by which autonomic neuropathy was defined were extracted to perform subgroup analysis. The senior author (UA) reviewed the combined extracted data to confirm the accuracy of data collection.

### Critical appraisal

A critical appraisal tool was used in the final studies to assess the risk of bias. The following tools were used to assess for risk of bias in cohort studies; Risk Of Bias In Non-randomized Studies - of Interventions (ROBINS-I) tool^13^ and randomised controlled trials; Risk-Of-Bias tool for randomized trials (RoB 2).^14^ Two authors (MC, AE) independently ascertained the risk of bias using the tools stated. The Food and Drug Administration (FDA) recommends a minimum of 2 years to obtain sufficient data in CVOT to assess cardiovascular risk in a controlled trial, therefore a 2-year duration was used as a predetermined cut-off for bias (less than 2 years representing short duration). Disagreements in the risk of bias score were resolved by the senior author (UA) where necessary. A sensitivity analysis was undertaken by eliminating studies at high risk of bias to ascertain if there was any effect on the result and subsequent conclusion.

### Meta-analysis and analysis of subgroups

A quantitative synthesis (meta-analysis) was undertaken using Review Manager (RevMan) software V.5.4. For the meta-analysis, adjusted relative risk (RR) were not presented by individual studies. Raw CVE and all-cause mortality data from CAN-positive and CAN-negative groups were collected. Subsequently, data were pooled across the studies with a random-effects method as moderate-high heterogeneity was expected. Statistical heterogeneity was assessed using the *I*^2^ statistic and % cut-offs for not important, moderate and substantial heterogeneity were in keeping with Cochrane guidance. An *a priori* decision was made not to present meta-analysis results where *I*^2^ >90%. Methodological variables (eg, definition of CAN) and clinical variables (eg, type of diabetes) were evaluated within subgroup analysis. The AFTs are summarized in online supplemental ESM table 2.

## Results

### Search results

After removing all duplicates, 18 467 articles were identified from the electronic database and manual reference searches of relevant systematic reviews. These titles and abstracts were screened using the prespecified inclusion/exclusion criteria, leading to the exclusion of 18 383 articles. Eighty-four articles were analysed for full-text eligibility, and subsequently 58 were excluded. Twenty-six papers fulfilled the inclusion criteria, and subsequent data were extracted (online supplemental ESM tables 3-5). Nine papers provided only CVE data, eight papers provided only mortality data and the remaining papers provided both CVE and mortality data.

### Study characteristics

#### Summary of settings

The majority of studies included were carried out in European populations (n=16). Other populations were Asian (n=5) and North American (n=5). CAN populations ranged from 19 to 941 participants.^15 16^ CAN-negative populations ranged from 17 to 1262 participants.^17 18^

#### Study design and participants

The majority of studies were prospective cohort studies (n=25), with one study being a case-control follow-up study. The mean age of participant groups varied from 33 years to 65.2 years.^17 19^ The majority of studies did not notably differ in the recruitment of participants based on sex. However, one study only recruited male participants in the CAN-negative group.^20^

Thirteen studies defined CAN as at least one positive AFT; however, three of these studies also had a subpopulation of CAN which required at least two positive AFTs. Seven studies required at least two positive AFTs to define CAN. One study required three or more positive AFTs to define CAN.^21^ For subgroup analyses, subpopulations of CAN requiring at least two positive AFTs from the three studies were included in the minimum two positive AFT subgroup. Out of the 26 studies included in the meta-analysis, five studies were not included in the subgroup analyses of the number of AFTs used to define CAN as the definition of CAN was either unavailable or unclear. One study did not state the required number of positive AFTs for CAN.^22^

#### Risk of bias

Assessment of the risk of bias in included studies is shown in online supplemental ESM table 6. Due to each study follow-up lasting longer than 2 years, there was a possibility of CAN development in the comparator group (CAN-negative) and thus resulting in bias due to deviation from the intended definitions; therefore, all trials had at least one component of the risk of bias.

### CAN and CVD risk

**Figure 1:**
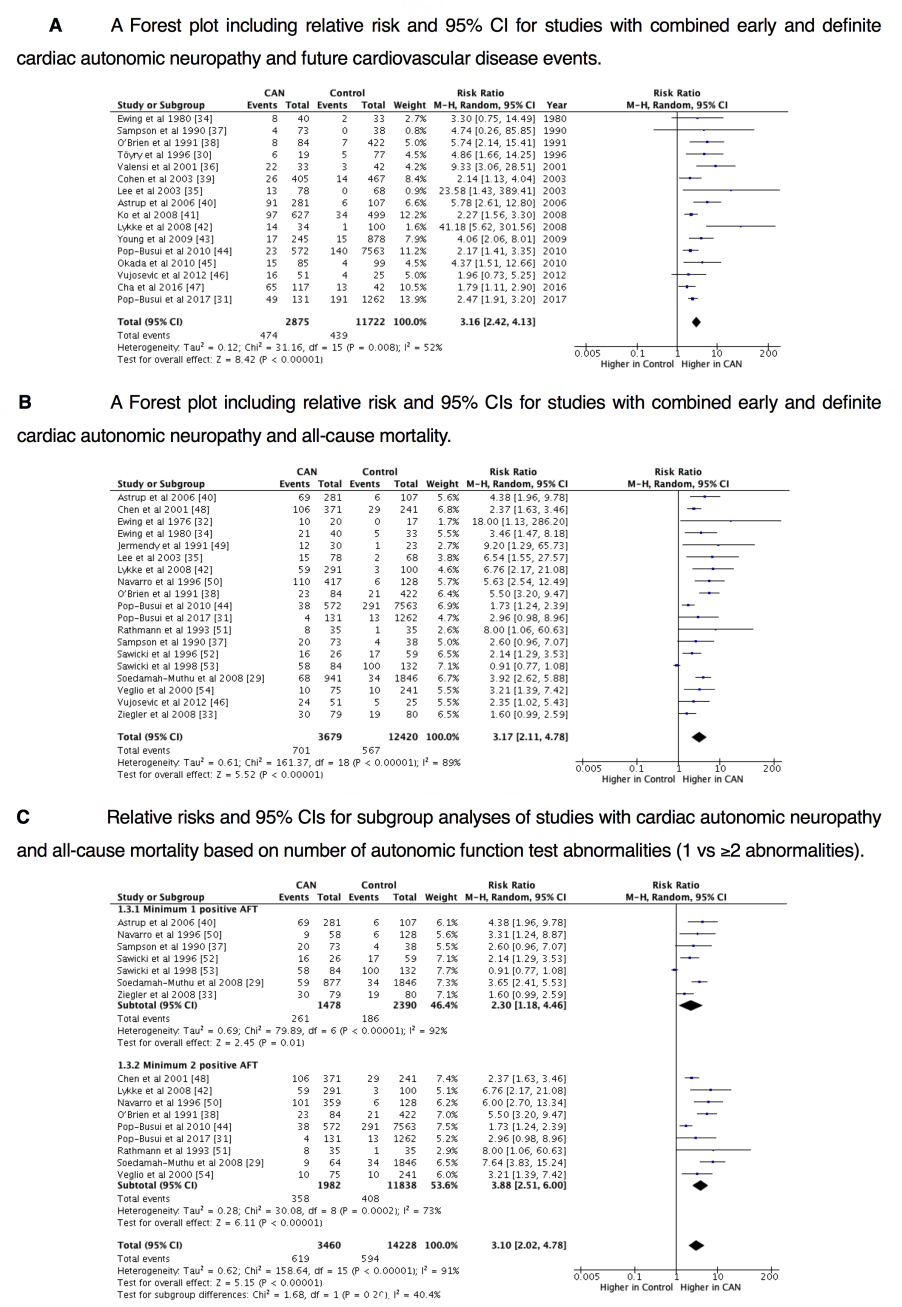
(A) A forest plot including relative risk (RR) and 95% CI for studies with combined early and definite cardiac autonomic neuropathy (CAN) and future cardiovascular disease events. (B) A Forest plot including RR and 95% CIs for studies with combined early and definite CAN and all-cause mortality. (C) RRs and 95% CIs for subgroup analyses of studies with CAN and all-cause mortality based on number of autonomic function test (AFT) abnormalities (1 vs ≥2 abnormalities).

Sixteen from the final 26 studies in the meta-analysis provided CVE data from participants with (n=2875) and without (n=11 722) CAN.^16 17 20–33^ Future CVE rate was higher for CAN at baseline in all individual studies, with significant differences between CAN-positive and CAN-negative cohorts in 13 studies. The pooled RR of future CVE in patients with CAN is demonstrated by the forest plot (figure 1A). The random effects Mantel-Haenszel estimate for the pooled RR for future CVE in patients with CAN was 3.16 (95%CI 2.42 to 4.13; p<0.00001, I^2^=52%).

### Subgroup analysis of CVD risk by diabetes subtype

Sixteen studies underwent subgroup analysis stratified according to the type of diabetes in the CAN population: type 1 or type two diabetes^16 17 21 23–33^ (online supplemental ESM figure 2). Five studies consisted of CAN population with only type 1 diabetes (n=603), which presented a RR for CVE of 5.54 (95%CI 2.28 to 13.45; p<0.0002, I^2^=76%).^17 23 24 26 28^ Nine studies consisted of CAN population with only type 2 diabetes (n=2199), with an RR of 2.45 (95%CI 1.93 to 3.11; p<00 001, I^2^=18%).^16 21 25 27 29–33^

### Subgroup analysis of CVD risk by number of positive AFTs

Twelve studies providing future CVE data underwent another subgroup analysis based on the number of positive AFTs used to define the presence of CAN; one positive AFT and minimum two positive AFTs^16 17 23–31 33^ (online supplemental ESM figure 3). Eight studies used minimum one positive AFT to define CAN (n=1783), which presented an RR for future CVE of 2.88 (95%CI 2.01 to 4.12; p<0.00001, I^2^=47%).^16 23 25–27 29 31 33^ Four studies used minimum two positive AFTs to define CAN (n=821),^17 24 28 30^ and one study had a subgroup with minimum two positive AFTs to define CAN (n=69),^33^ which resulted in an RR of 2.84 (95%CI 1.84 to 4.38; p<0.00001, I^2^=67%).

### CAN and all-cause mortality risk

Nineteen studies included provided mortality data from patients with (n=3679) and without (n=12 420) CAN.^15 17–21 23 24 26 28 30 32 34–40^ Except for one study, all-cause mortality rates were higher in patients with CAN than in patients without CAN, with significant differences in 15 studies in CAN groups.

The random effects Mantel-Haenszel estimate for the pooled RR for all-cause mortality in patients with CAN was 3.17 (95%CI 2.11 to 4.78; p<0.00001, I^2^=89%). The RR of all-cause mortality based on CAN presence/absence is shown by the forest plot (figure 1B). Mean follow-up intervals of all 19 studies ranged from 2.75 years to 10.1 years.^18 26^

### Subgroup analyses of CAN and all-cause mortality risk by diabetes subtype

Fourteen studies providing mortality data underwent subgroup analysis based on the type of diabetes in the CAN population; type 1 or type 2 diabetes^15 17 21 23 24 26 28 30 32 34 36 38–40^ (online supplemental ESM figure 4). Nine studies consisted of CAN population with only type 1 diabetes (n=2319), which presented an RR for all-cause mortality to be 3.76 (95%CI 2.89 to 4.91; p<0.00001, I^2^=23%).^15 17 23 24 26 28 36 38 40^ Five studies consisted of CAN population with only type 2 diabetes (n=1156), with an RR of 1.94 (95%CI 1.03 to 3.65; p=0.04, I^2^=92%).^21 30 32 34 39^ High heterogeneity in the type 2 diabetes subgroup may be due to the number of positive AFTs used to define CAN; one study used minimum three positive AFTs,^21^ two studies used minimum two positive AFTs,^30 34^ one study used minimum one positive AFT^39^ and one study had an unclear CAN definition.^32^

### Subgroup analyses of CAN and all-cause mortality risk by number of positive AFTs

Fourteen studies providing mortality data underwent another subgroup analysis based on the number of positive AFTs used to define the presence of CAN; one positive AFT and minimum two positive AFTs^15 17 19 23 24 26 28 30 34 36–40^ (figure 1C). Seven studies used one positive AFT to define CAN (n=1478) with an RR of all-cause mortality of 2.30 (95%CI 1.18 to 4.46; p=0.01, I^2^=92%).^15 19 23 26 36 38 39^ All barring one study consisted of CAN populations with type 1 diabetes, with that sole study presenting the only RR <1, suggesting a possible reason for heterogeneity.^39^ Seven studies used minimum two positive AFTs to define CAN (n=1559),^17 24 28 30 34 37 40^ with two studies presenting a subgroup using minimum two positive AFTs to define CAN (n=423)^15 36^ with an RR of 3.88 (95%CI 2.51 to 6.00; p<0.00001, I^2^=73%).

### Sensitivity analyses

A sensitivity analysis was undertaken after removing three studies at high risk of bias, as shown by the forest plots in online supplemental ESM figures 5,6. The analysis showed a similar RR for both CVE (RR: 3.05, 95% CI 2.32 to 4.00; p<0.0001, I^2^=52%), and all-cause mortality (RR: 2.81, 95% CI 1.85 to 4.27; p<0.00001, I^2^=88%). This indicates that the primary analysis and conclusion remain robust.

## Discussion

We found a significant association between early and definite CAN and CVE in patients with diabetes. The risk of CVE and mortality was greater in type 1 compared with type 2 diabetes and with definite compared with early/possible CAN. CAN therefore remains a target for prevention of both development and subsequent progression.

In keeping with our study, previous data have demonstrated that the presence of CAN results in a 5-year mortality rate which is three times greater compared with that seen in those without CAN.^24^ Previous studies such as the European Diabetes (EURODIAB) Prospective Cohort Study (n=2787) and the Action to Control Cardiovascular Risk in Diabetes (ACCORD) Trial confirmed the association of CAN and mortality in patients with type 1 and type two diabetes, respectively.^30^ ^15^ The ACCORD Trial also adopted a successful approach for CAN prevention, where a negative CVD history identified patients who benefitted from an intensive glycemic control.^41^ Such an approach may be adopted in wider clinical practice.^42^

In a seminal study, Ewing *et al* demonstrated a 2.5-year mortality rate of 27.5% that increased by 25.5% after 5 years in patients with diabetes and definite CAN^20^ which is in contrast to patients with diabetes and a normal AFT who had a mortality rate of only 15% over the same 5-year period.^20^ CAN also prognosticates for CVE and mortality in the presence of intensive glycemic control in type 2 diabetes, as demonstrated by the Action in Diabetes and Vascular Disease: Preterax and Diamicron Modified Release Controlled Evaluation (ADVANCE), Veterans Affairs Diabetes Trial (VADT) and ACCORD Studies.^8 30 43–45^ The excess of CVE may be partly explained by an association between CAN and reduced myocardial flow reserve in type 1 diabetes.^46^ Similar data have also been demonstrated in type 2 diabetes.^47^ Myocardial flow reserve is a strong predictor of cardiac mortality and non-fatal myocardial infarction in patients with diabetes.^46^

In a post hoc analysis of two large cohort studies with stable and chronic CVD participants, the ONgoing Telmisartan Alone and in combination with Ramipril Global Endpoint Trial and the Telmisartan Randomized Assessment Study in ACE intolerant subjects with cardiovascular disease Trial (ONTARGET/TRANSCEND Studies), significant increases in CVE and all-cause mortality were independently associated with increased resting and baseline heart rate (HR).^48^ Importantly, the ADVANCE Study demonstrated an increase of 10 beats/min in resting and average HR leading to a significant increase in risk of cardiovascular death and all-cause mortality (adjusted hazard ratio 1.15 per 10 bpm).^49^ High resting HR and blunted HRV are both indicators of cardiac autonomic nervous system abnormalities, the impact of which were assessed in the Framingham Heart Study offspring cohort.^50^ Two studies reported presence of SMI, both of which showed an increased frequency in the presence of CAN.^17 22^ A meta-analysis of 12 cross-sectional studies (n=1468) demonstrated a significant association between CAN and the presence of SMI with a pooled prevalence RR of 1.96 (95%CI 1.53 to 2.51; p<0.001) (in favour of CAN).^5^

Other studies demonstrate that diabetic neuropathy and nephropathy are also associated with increased mortality risk, although these studies typically do not adjust for CAN.^51^ ^52^ However, the relationship between the different microvascular complications is complex as peripheral neuropathy has been shown to predict CKD in type 2 diabetes mellitus (T2DM).^53^ For instance, in a large meta-analysis (n=1 28 505) there was an association between decreased estimated glomerular filtration rate (eGFR), albuminuria and mortality; the presence of diabetes did not alter this association with either CVE or mortality.^51^ CAN is a demonstrable risk factor for CKD in patients with type 1 and 2 diabetes.^54^ CAN is known to impair sympathetically mediated dilation of coronary resistance vessels,^55^ and thus has a mechanistic cause for the excess CVE and mortality exhibited.

Early detection of CAN is key to the success of therapeutic response, as it has been suggested that cardiovascular denervation may be reversible if multifactorial and lifestyle interventions are implemented soon after onset.^56^ The American Diabetes Association position statement for diabetic neuropathy advocates the prevention of CAN. Indeed, in the Intensified Multifactorial Intervention in Patients With Type 2 Diabetes and Microalbuminuria (STENO-2) Trial, multifactorial intervention was beneficial for autonomic neuropathy (OR: 0.32, 95% CI 0.12 to 0.78 for intensive therapy vs standard therapy)^57^ and persisted beyond two decades.^57^ Multiple studies have shown that autonomic balance can be reinstated using diet and exercise lifestyle measures, possibly reversing CAN. The Diabetes Prevention Program Trial (n=2980) demonstrated that lifestyle modification improved heart rate, HRV, and QT interval in prediabetes, with a significant improvement on metformin in most of these parameters. In a study of type 2 diabetes with obesity of differing low energy diets over an 8-week intervention period, attainment of a median 1198 kJ daily decrease in total energy intake (weight loss of 5–6 kg) improved HRV and parasympathetic nervous system function irrespective of the diet.^58^ A number of studies have evaluated the outcomes of weight loss with bariatric surgery or calorie restriction in individuals with diabetes, demonstrating improvements in parasympathetic indices of HRV alongside an improved sympathovagal balance. Aerobic exercise training programs implemented in patients with type 2 diabetes three times a week for 6 months also demonstrates significant improvement in HRV indices^59^ with the greatest improvement in definite CAN, with a 40% reduction in low frequency power compared with controls without CAN.^59^ Furthermore, in those with obstructive sleep apnoea (OSA), long-term continuous positive airway pressure therapy can significantly improve cardiac autonomic function, measured using spectral indices of HRV that include compensation for changes in breathing pattern.^60^

Early intervention with intensive control of glycemia in patients with type 1 diabetes helps to reduce the progression and development of CAN. When considering CAN in type 1 diabetes, the SEARCH CVD study evaluated subclinical autonomic dysfunction in 354 young patients with type 1 diabetes. HRV testing was used to evaluate dysfunction and the occurrence of parasympathetic loss with sympathetic override. HbA1c>59 mmol/mol (>7.5%) was independently associated with the incidence of subclinical CAN when compared with patients without diabetes.^61^ The Diabetes Control and Complications Trial (DCCT) demonstrated that intensive glycemic control decreased CAN incidence by half in type 1 diabetes over a follow-up of 6.5 years, when compared with conventional therapy (7% vs 14%, p<0.004).^62^ During long-term follow-up in the Epidemiology of Diabetes Interventions and Complications (EDIC) Study, this beneficial association with the legacy of intensive hyperglycemic control persisted, with enduring benefits.^63^

A number of studies of sodium-glucose transport protein 2 (SGLT2) inhibitors have evaluated cardiovascular outcomes. The Empagliflozin Cardiovascular Outcome Event Trial in Type 2 Diabetes Mellitus Patients–Removing Excess Glucose (EMPA-REG) outcome trial^64^ demonstrated a modest blood pressure reduction without an increase in HR signaling a reduction in sympathetic tone. It has been postulated that SGLT2 inhibitors may exert a beneficial effect by reducing sympathetic nervous system overactivity, subsequently reducing cardiovascular risk and the development of nephropathy in diabetes.^65^ Furthermore, a recent meta-analysis (n=52 115) showed a lower risk of AF (RR: 0.82, 95% CI 0.70 to 0.96) and ventricular tachycardia (RR: 0.73, 95% CI 0.53 to 0.99) with SGLT2 inhibitor use.^66^ Prospective cohort studies and randomized controlled trials of SGLT2 inhibitors are required to evaluate any efficacy in the reduction in CAN prevalence or improvement in autonomic dysfunction.

Our study was reported in accordance with PRISMA guidelines, the gold standard in reporting systematic reviews. The large number of cohort studies involved in the meta-analyses also provides strong causal evidence for the relationship between CAN and CVE and mortality. Limitations include variation in CAN definition (1 AFT vs 2 AFT positive). However, this area of heterogeneity was studied by subgroup analysis. Additionally, only data from English language publications were obtained. Unfortunately, adjusted RR with 95% CI could not be included due to a lack of presented data within the manuscript in the original articles limiting the analysis of confounding factors, for example, diabetic nephropathy and underlying CVD disorders. Some demographics and comorbidities data were not available within all articles; therefore, secondary analysis was limited.

### Conclusion

CAN is a major prognostic indicator of CVE and mortality and as such simple, quick and non-invasive testing for autonomic dysfunction should be incorporated into population screening, especially considering its preventability and reversibility with lifestyle and pharmacological interventions. This may beneficially address the high residual CV risk we observe in our patients with diabetes even with contemporary treatments and approaches.

## Data Availability

All data relevant to the study are included in the article or uploaded as supplementary information.
