# Supplementary material for: Cardiac autonomic neuropathy and risk of cardiovascular disease and mortality in type 1 and type 2 diabetes: a meta-analysis
Source: BMJ Open Diabetes Res Care. 2021 Dec 30;9(2):e002480. doi: 10.1136/bmjdrc-2021-002480 (PMC8719152; doi:10.1136/bmjdrc-2021-002480)

ESM – Extended supplementary material

ESM Table 1 Search terms

Cardiac autonomic neuropathy (CAN)	Cardiovascular disease events	Mortality
• Diabetic neuropathy [MeSH Terms]<br>• Autonomic nervous system diseases [MeSH Terms]<br>• Cardiac autonomic neuropathy<br>• Cardiovascular autonomic dysfunction<br>• Autonomic neuropathy<br>• Abnormal heart rate variability<br>• Autonomic Function Tests<br>• Cardiac autonomic function tests<br>• Cardiovascular autonomic reflex tests<br>• Severe cardiac autonomic neuropathy<br>• 30:15 ratio<br>• E/I<br>• E:I<br>• Valsalva ratio<br>• Heart rate response to deep breathing<br>• Resting heart rate variability<br>• Sympathetic nervous system overactivity<br>• SNS overactivity	• Heart Arrest[MeSH Terms]<br>• heart failure[MeSH Terms]<br>• Heart Failure [MeSH]<br>• embolism[Mesh]<br>• myocardial ischemia[MeSH Terms]<br>• Brain Ischemia[Mesh]<br>• Intracranial Embolism and Thrombosis[Mesh]<br>• thromboembolism [Mesh]<br>• thrombosis [Mesh]<br>• Stroke[Mesh]<br>• CVD<br>• CHD<br>• NSTEMI<br>• Cerebrovascular accident<br>• Brainstem<br>• Thrombosis<br>• Heart attack<br>• Silent Myocardial ischaemia<br>• Silent myocardial infarction<br>• PCI	• Mortality<br>• Cardiovascular mortality<br>• Perioperative mortality<br>• All-cause mortality

Chowdhury M, *et al. BMJ Open Diab Res Care* 2021; 9:e002480. doi: 10.1136/bmjdrc-2021-002480

• Vagus nerve denervation<br>• Sudomotor function<br>• R-R interval variation<br>• Diabetic neuropathy<br>• Diabetic autonomic neuropathy	• Percutaneous coronary intervention<br>• MACE<br>• Perioperative myocardial infarction<br>• Cardiovascular disease<br>• Stroke<br>• Brain ischemia<br>• Vascular<br>• STEMI	

ESM Table 2 Tests for autonomic function

Autonomic function tests (AFT)
• Heart-rate response to the Valsalva manoeuvre (Valsalva ratio)<br>• Heart-rate response to deep breathing shown by the Expiratory/Inspiratory (E/I) ratio<br>• Heart-rate response to standing at 30 seconds and 15 seconds (30:15 ratio)<br>• BP response to standing (mmHg)<br>• BP response to sustained handgrip (mmHg)<br>• Heart rate variation (HRV)<br>• Spectral power analysis (of high frequency, very low frequency and low-frequency bands)

ESM Table 3 Participant demographic characteristics information from all final selected articles

Study	Country	Study design	Type of diabetes	Age (years)	Gender (M/F)
Chen et al, 2001(32)	Taiwan	Cohort study	Type 2	CAN-: 61.3 +/- 11.4<br><br>CAN+: 64.2 +/- 10.1	CAN-: 171/70 (71%/29%)<br><br>CAN+: 260/111 (70%/30%)
Ko et al, 2008 (25)	Korea	Cohort study	Type 2	Mean age of total study population: 58.1+/-10.7	Total study population: 431/695 (38%/62%)
Pop-Busui et al, 2017 (15)	America and Canada	Cohort study	Type 1	CAN-: 33+/-7.0<br><br>CAN+: 37+/-7.0	CAN-: 674/588 (53%/47%)<br><br>CAN+: 61/70 (47%/53%)
Vujosevic et al, 2012 (30)	Montenegro	Cohort study	Type 2	Mean age of the total study population: 64.08+/-9.06	Total study population: 43/33 (57%/43%)
Valensi et al, 2001 (20)	France	Cohort study	Type 1 and Type 2	Mean age of the total study population: 54.7 (range 30.0-70.0)	NA
Soedamah-muthu et al, 2008 (13)	31 centres in 16 European countries	Cohort study	Type 1	Mean age of the total study population: 33.0 (range 15.0-61.0)	Total study population: 1421/1366 (51%/49%)
O'Brien et al, 1991 (22)	England	Cohort study	Type 1	CAN-: 45.0+/-18.0<br><br>CAN+: 46.0+/- 15.0	CAN-: 245/167 (59%/41%)<br><br>CAN+: 36/48 (43%/57%)
Lee et al, 2003 (19)	Korea	Cohort study	Type 2	CAN-: 58.0+/-10.0<br><br>CAN+: 65.0+/-8.0	NA
Ewing et al, 1980 (18)	Scotland	Cohort study	NA	Mean age of the total study	CAN-: 33/0 (100%/0%)

				population: 46.1 (range 24.0-69.0)	CAN+: 29/11 (73%/27%)
Cha et al, 2016 (31)	South Korea	Cohort study	Type 2	Mean age of the total study population: 62.5+/- 8.7	Total study population: 65/94 (41%/59%)
Astrup et al, 2006 (24)	Denmark	Cohort study	Type 1	Diabetic nephropathy group: 41.0+/-9.0<br><br>Without diabetic nephropathy group: 43.0+/-10.0	Total study population: 237/151 (61%/39%)
Lykke et al, 2008 (26)	Denmark	Cohort study	Type 1	Normoalbuminuric group (n=192): 42.7+/-10.2<br><br>Nephropathy group (n=199): 40.9+/-9.6	Total study population: 240/151 (61%/39%)
Cohen et al, 2003 (23)	America	Cohort study	Type 2	Stroke (endpoint) group: 62.3+/-6.9<br><br>no stroke (endpoint) group: 58.4+/-8.4	Male stroke (endpoint) group: 24/17 (59%/41%)<br><br>No stroke (endpoint) group: 555/354 (61%/39%)
Okada et al, 2010 (29)	Japan	Cohort study	Type 2	Preserved BRS = 55.6+/-10.5<br><br>Depressed BRS = 60.6+/-12.0	Preserved BRS: 63/36 (64%/36%)<br><br>Depressed BRS: 31/54 (36%/64%)<br><br>Total study population: 94/90 (51%/49%)
Navarro et al, 1996 (34)	America	Cohort study	Type 1	Mean age of the total study population: 33.4+/-9.0	Total study population: 244/301 (45%/55%)

Sampson et al 1990 (21)	England	Cohort study	Type 1	Group A: 37.1+/-1.2<br><br>Group B: 39.5+/-1.7<br><br>Group C: 35.5+/-1.3	Group A: 23/26 (47%/53%)<br><br>Group B: 10/14 (42%58%)<br><br>Group C: 22/16 (58%/42%)
Töyry et al, 1996 (14)	Finland	Cohort study	Type 2	NA	Total study population: 70/63 (53%/47%)
Veglio et al, 2000 (38)	Italy	Cohort study	Type 1	NA	Total diabetic population: 196/183 (52%/48%)
Ziegler et al, 2008 (17)	Germany	Cohort study	NA	Mean age of the total study population: 65.2+/-5.5	Total diabetic population: 82/78 (51%/49%)
Sawicki et al, 1996 (36)	Germany	Cohort study	Type 1	Mean age of the total study population: 40.0+/-11.0	Total study population: 50/35 (59%/41%)
Young et al, 2009 (27)	14 centres in the United States and Canada	Cohort study	Type 2	No screening (n=562) group: 60.8 +/-6.4<br><br>Screening (n=561) group: 60.7 +/-6.7	No screening (n=562) group: 311/251 (55%/45%)<br><br>Screening (n=561) group: 290/271 (52%/48%)
Sawicki et al, 1998 (37)	Germany	Case-control follow up study	Type 2	Median age of the total study population: 63.0 (interquartile range 54.0–71.0)	Total study population: 68/148 (31%/69%)

Pop-Busui et al, 2010 (28)	America and Canada	Cohort study	Type 2	CAN1: 62.5+/-6.7<br><br>CAN-: 61.9+/-6.7	CAN1: 288/284 (50%/50%)<br><br>CAN-: 4606/2957 (61%/39%)
Rathmann et al, 1993 (35)	Germany	Cohort study	Type 1 and type 2	CAN-: 42.9+/-12.0<br><br>CAN+: 43.3+/-12.0	CAN-: 12/23 (34%/66%)<br><br>CAN+: 12/23 (34%/66%)
Ewing et al, 1976 (16)	Scotland	Cohort study	NA	Mean age of the total study population: 47.0 (range 24.0-63.0)	31/6 (84%/16%) initially. 30 patients were re-examined 18 months to 2 years after initial selection for CAN, but sex was not available for them.
Jermendy et al, 1991 (33)	Hungary	Cohort study	Type 1 and type 2	Mean age of diabetic population: 46.3+/-1.7	Total diabetic population: 35/18 (66%/34%)

*CAN, cardiac autonomic neuropathy; CAN+, cardiac autonomic neuropathy positive; CAN-, cardiac autonomic neuropathy negative; NA, information not available

ESM Table 4 Participant cardiovascular disease event and mortality rates

Study	Follow-up (years)	AFT method of assessment	Definition of CAN	Cardiovascular disease events definition	Cardiovascular disease events % (CAN +)	Cardiovascular disease events % (CAN -)	Mortality % (CAN +)	Mortality % (CAN -)
Chen et al, 2001 (32)	7.7	1) Heart rate response to a single deep breath.<br><br>2) Heart rate response to 6 consecutive breaths.<br><br>3) Heart rate response to standing.<br><br>4) Blood pressure response to standing.<br><br>5) Heart rate response to Valsalva manoeuvre.	Defined by scoring 3 or more following unique diagnostic criteria	NA	NA	NA	106/371 (29%)	29/241 (12%)
Ko et al, 2008 (25)	7.1	1) E/I ratio<br><br>2) 30:15 ratio<br><br>3) Heart rate response to Valsalva manoeuvre	Defined based on at least one abnormal standard test	1)New onset of ischaemic stroke	97/627 (15%)	34/499 (7%)	NA	NA

Pop-Busui et al, 2017 (15)	3–9 years (mean 6.5 years). In 1994, 96% of the surviving DCCT cohort enrolled in the EDIC observational study in an additional 20 years of follow-up.	1) R-R variation in response to paced breathing<br><br>2) Valsalva manoeuvre<br><br>3) Postural changes in blood pressure	Defined as either an R-R variation <15 or an R-R variation 15-19.9 in combination with a Valsalva ratio <=1.5 or a decrease of >10 mmHg in diastolic blood pressure upon and while standing for 10 min.	1)Nonfatal myocardial infarction or stroke<br><br>2) death judged to be secondary to CVE<br><br>3) subclinical ("silent") myocardial infarction<br><br>4)Angina confirmed by ischemic changes with exercise tolerance testing or by clinically significant obstruction on coronary angiography<br><br>5)Congestive heart failure with paroxysmal nocturnal dyspnoea, orthopnoea, or marked limitation of physical activity caused by heart disease	49/131 (37%)	191/1262 (15%)	4/131 (3%) *death from cardiovascular diseases only	13/1262 (1%) *death from cardiovascular diseases only

				6)Revascularization with angioplasty and/ or coronary artery bypass.<br><br>7)Major adverse cardiovascular events (MACE) defined as nonfatal myocardial infarction or stroke or CVD death.				

Vujosevic et al, 2012 (30)	13<br><br>*CAN (n=51) and control (n=25) population was 76 in total intrahospital, however 8 died intrahospital and it isn't stated how many were CAN patients vs control. Therefore, cannot work out how many CAN vs control patients were part of the extra hospital 10 year follow-up.	1)Heart rate responses to the Valsalva manoeuvre<br><br>2)Standing up and deep breathing<br><br>3)Blood pressure responses to standing up<br><br>4)Blood pressure response to sustained handgrip	Defined as normal (all tests normal), early (one heart rate test abnormal), definite (two or more heart rate tests abnormal), severe (abnormal heart rate tests plus one or both blood pressure tests abnormal), or atypical (any other combination of abnormalities).	NA	16/51 (31%)<br>*only includes cardiovascular events that led to death	4/25 (16%)<br>*only includes cardiovascular events that led to death	24/51 (47%)	5/25 (20%)

Valensi et al, 2001 (20)	4.5	1) Heart rate response to the Valsalva manoeuvre<br><br>2) Heart rate response to 6 consecutive breaths<br><br>3) Heart rate response to standing	The results of the three tests were compared with those from a control series with age taken into account.	1)Death of cardiac origin (sudden death or death caused by MI or congestive heart failure)<br><br>2)Nonfatal MI (MI was considered to be a major event whether the patient was hospitalized or not)<br><br>3)Heart failure<br><br>4)Resuscitation from ventricular tachycardia/fibrillatio n<br><br>5)Need for coronary revascularization.	22/33 (67%)	3/42 (7%)	NA	NA

Chowdhury M, *et al. BMJ Open Diab Res Care* 2021; 9:e002480. doi: 10.1136/bmjdrc-2021-002480

Soedamah-Muthu et al, 2008 (13)	7	1)Loss of heart rate variability<br><br>2)Postural hypotension	1) Loss of heart rate variability with an RR ratio of <1.04 and/or postural hypotension with a fall in systolic blood pressure of >=20 mmHg).<br><br>2)At least two abnormal tests with a RR ratio of <1.04 and postural hypotension with a fall in systolic blood pressure of >=30 mmHg.	NA	NA	NA	CAN definition 1: 59/877 (7%)<br><br>CAN definition 2: 9/64 (14%)	34/1846 (2%)

O'Brien et al, 1991 (22)	5	HRV in response to<br><br>1)Supine rest<br><br>2)Single deep breath<br><br>3)Valsalva manoeuvre<br><br>4)Standing for 60 seconds	Defined as heart rate responses below the 2.5th centile in two or more of the four tests.	NA	NA	NA	23/84 (27%)	21/422 (5%)

Lee et al, 2003 (19)	3.8	1)Deep breathing tests were performed at 6 ventilations/min (abnormal RR-interval ratio <1.11)<br><br>2)Valsalva manoeuvre were done by having the patient blow at 40 mm Hg for 15 seconds (abnormal RR-interval ratio <1.21)<br><br>3)Lying-to-standing tests were performed by having the patient move from the standing to the recumbent position (abnormal RR-interval ratio <1.04).<br><br>4)Postural systolic blood pressure change was measured with the patient in the recumbent position and again after the patient had been in the standing position for 30 seconds (abnormal blood pressure decrease >30 mm Hg)	Defined as the presence of 3 or more abnormal test results.	1)Major cardiac events were defined as either cardiac death as confirmed by review of hospital records<br><br>2)Nonfatal myocardial infarction as evidenced by the appropriate combination of symptoms, electrocardiographic study results and enzyme changes.	13/78 (17%)	0/68 (0%)	15/78 (19%)	2/68 (3%)

		5)Handgrip tests were measured as the increase in diastolic blood pressure during 30% maximal handgrip (abnormal blood pressure increase <10 mm Hg).						
Ewing et al, 1980 (18)	1.6 – 5	1)Heart rate responses to the Valsalva manoeuvre<br><br>2)Blood pressure response to sustained handgrip<br><br>3)Postural fall in blood pressure	Defined as either one or both responses to the Valsalva manoeuvre and sustained handgrip were abnormal, or if both were borderline. Patients with one borderline autonomic function test were designated as 'normal'.	NA	8/40 (20%) *only cardiovascular events leading to death mentioned	2/33 (6%) *only cardiovascular events leading to death mentioned	21/40 (53%)	5/33 (15%)

Cha et al, 2016 (31)	8.9	1)E/I ratio<br><br>2)Responses to the Valsalva manoeuvre<br><br>3)HR response from lying to standing.	Each measurement was scored as normal = 0 or abnormal = 1<br><br>Defined as<br><br>1)Normal autonomic function = 0<br><br>2)Early CAN = 1<br><br>3) Definite CAN >= 2	1) Recurrent attack of CVE, which was defined as CVE (MI, non-MI acute coronary syndrome, heart failure, or death attributable to CVE), stroke or limb amputation from diabetic foot, according to World Health Organization (WHO) criteria.	Early CAN: 22/48 (46%)<br><br>Definite CAN: 43/69 (62%)<br><br>*2 lower limb amputations included in CVE but does not state if part of CAN population or control	13/42 (31%)<br><br>*2 lower limb amputations included in CVE but does not state if part of CAN population or control	NA	NA

Astrup et al, 2006 (24)	10.1 years	1)Expiration/Inspiration (E/I) variation in heart rate.	Defined as<br><br>1) Abnormal: HRV <=10 bpm<br><br>2) Borderline: HRV 11–14 bpm<br><br>3) Normal: HRV >=15 bpm	1) Cardiovascular mortality and morbidity. Cardiovascular morbidity was defined as a history of nonfatal myocardial infarction, percutaneous coronary intervention, coronary artery bypass grafting, nonfatal stroke, amputation as a result of ischemia, and vascular surgery for peripheral atherosclerotic disease.	Abnormal HRV: 85/216 (39%)<br><br>Borderline HRV: 6/65 (9%)<br><br>*37 total Lower limb amputation /peripheral bypass procedures included in abnornal/borderline/normal HRV group however not stated how many in each groups	6/107 (6%)<br><br>*37 total Lower limb amputation /peripheral bypass procedures included in abnornal/borderline/normal HRV group however not stated how many in each groups	Abnormal HRV:62/216 (29%)<br><br>Borderline HRV:7/65 (11%)<br><br>*37 total Lower limb amputation/peripheral bypass procedures included in abnornal/borderline/normal HRV group howev	6/107 (6%)<br><br>*37 total Lower limb amputation/peripheral bypass procedures included in abnornal/borderline/normal HRV group however not stated how many in each groups

							er not stated how many in each groups	
Lykke et al, 2008 (26)	10	1)Heart rate response to deep breathing<br><br>*RR and QT intervals were also measured in all leads in three consecutive periods and a mean value for each was calculated.	NA	NA	Combined HRV and QTc (abnormal) : 14/34 (41%)	Combined HRV and QTc (normal): 1/100 (1%)	Combined HRV and QTc (abnormal): 15/34 (44%)<br><br>Combined HRV and QTc (borderline):44/257 (17%)	Combined HRV and QTc (normal) : 3/100 (3%)

Cohen et al, 2003 (23)	5.3	1)E/I ratio	Defined as normal, abnormal or borderline based on age-related range values (Smith, 1982).	1) Death due to cardiovascular events (sudden death, progressive heart failure, fatal myocardial infarction, fatal arrhythmias, cerebral vascular accidents and ruptured aortic aneurysm)<br><br>2)Non-fatal myocardial infarction<br><br>3)Non-fatal cerebral vascular accident<br><br>4)Heart failure requiring hospital admission<br><br>5)Pulmonary infarction.	26/405 (6%)<br><br>*All strokes	14/467 (3%)<br><br>*All strokes	NA	NA

Okada et al, 2010 (29)	4.7	1) Baroreceptor sensitivity (BRS) was assessed using the phenylephrine method. BRS was calculated as the slope of the linear regression line relating the systolic BP changes to the RR interval changes.	NA	1)Major adverse cardiovascular event (MACE), which included cardiovascular mortality, nonfatal MI, coronary revascularization by angioplasty or bypass, stroke and congestive heart failure requiring admission.	Depressed BRS: 15/85 (18%)	Preserved BRS: 4/99 (4%)	NA	NA
Navarro et al, 1996 (34)	1 – 11.5	1)Heart rate was monitored continuously during slow breathing at 6/min. The averaged difference between the highest heart rate with inspiration and the lowest rate with expiration during seven consecutive breathing cycles was called the AR6.<br><br>2)Valsalva ratio<br><br>(Normal limits were established for the age group examined (AR6 >= 15.0; VR >= 1.43).	Defined as<br><br>1)CRR 0: both tests normal<br><br>2)CRR 1: one test abnormal<br><br>3)CRR 2: two tests abnormal	NA	NA	NA	CRR 1: 9/58 (16%)<br><br>CRR 2: 101/359 (28%)	6/128 (5%)

Sampson et al, 1990 (21)	10 – 15	1)Initially at this time the heart rate variability on deep respiration at six cycles/min, was the only test in common use.<br><br>The repeat tests of autonomic function were the heart rate variability on deep breathing at six cycles/min, the heart rate responses to the Valsalva manoeuvre (Valsalva ratio) and the heart rate and systolic blood pressure responses to standing as described elsewhere.	Group A: Early symptomatic autonomic neuropathy and an abnormal HRV (5.6 +/- 0.5)<br><br>Group B: Abnormal HRV (7.6 +/-0.5) alone<br><br>Group C: An asymptomatic control group with a normal HRV of 16-26 (20.8 +/- 0.5)	NA	Group A:4/49 (8%)<br><br>Group B:0/24 (0%)	Group C:0/38 (0%)	Group A:18/49 (37%)<br><br>Group B:2/24 (8%)	Group C:4/38 (11%)
Töyry et al, 1996 (14)	5, 10<br><br>*Stroke only assessed at 10 year follow-up	1) E/I ratio (Parasympathetic test at baseline and at 5-year examination)<br><br>2) Blood pressure response to standing (Sympathetic test at 5-year examination)	NA	1)First strokes	Parasympathetic neuropathy : 6/19 (32%)	No Parasympathetic neuropathy : 5/77 (6%)	NA	NA

Veglio et al, 2000 (38)	5	1) Heart rate response to deep breathing<br><br>2) Blood pressure response to standing<br><br>3)Heart rate (resting)	Defined as 2 or more abnormal results for the cardiovascular tests and heart rate	NA	NA	NA	10/75 (13%)	10/241 (4%)
Ziegler et al, 2008 (17)	9	1) Time domain measures including the standard deviation of R-R intervals (SDNN), coefficient of variation (CV) of R-R intervals, and the difference between the maximum and minimum R-R interval (max-min difference).	NA	NA	max-min R-R interval difference at the first quartile: 30/79 (38%)	max-min R-R interval difference at the 2nd-4th quartile: 19/80 (24%)	NA	NA
Sawicki et al, 1996 (36)	9	1) RR variation between supine and standing position	Defined as RRsupine/RRstanding below 1.03	NA	16/26 (62%)	17/59 (29%)	NA	NA

Young et al, 2009 (27)	4.8	1) Heart rate response to standing	Defined as lowest quartile of heart rate response to standing test	1)Nonfatal myocardial infarction<br><br>2)Cardiac death (fatal myocardial infarction within 30 days, death due to heart failure, arrhythmia or sudden cardiac death)	17/245 (7%)	15/878 (2%)	NA	NA
Sawicki et al, 1998 (37)	15 – 16	1) RR variation between supine and standing position	Defined as RRsupine/RRstanding below 1.03	NA	NA	NA	58/84 (69%)	100/132 (76%)

Pop-Busui et al, 2010 (28)	3.5	1)Resting heart rate<br><br>2)SD of normally conducted R-R intervals (SDNN).<br><br>*From simultaneous lead recordings, QT intervals were measured, and the QT index (QTI) was calculated as observed/ predicted QT duration where predicted value was based on Bazett's correction (QTc = QT/R - R^1/2).	Defined as<br><br>1)CAN1: lowest quartile of SDNN (<7.815 ms) and the highest quartile of QTI (>104.32%)<br><br>2)CAN2: lowest quartile of SDNN and the highest quartiles of QTI and resting heart rate<br><br>3)CAN3: lowest quartile of SDNN and the highest quartiles of QTI and heart rate, in the presence of diabetic peripheral neuropathy .<br><br>*CAN1 was the definition used for our meta-analysis	1)Death from CVE included deaths from myocardial infarction, heart failure, arrhythmia, invasive cardiovascular interventions, cardiovascular causes after non-cardiovascular surgery, stroke, unexpected death presumed to be from ischemic CVE occurring within 24 h after the onset of symptoms, and death from other vascular diseases.	CAN1: 23/572 (4%)<br><br>*Cardiovascular mortality	140/7563 (2%)<br><br>*Cardiovascular mortality	CAN1: 38/572 (7%)	291/7563 (4%)

Rathmann et al, 1993 (35)	8	1)Heart rate variation at rest<br><br>2)Heart rate variation at deep breathing	Defined as both parameters below two standard deviations of an age-corrected mean value of a non-diabetic control group	NA	2/35 (6%)<br><br>*death by myocardial infarction and stroke	1/35 (3%)<br><br>*death by cardiac arrhythmia	8/35 (23%)	1/35 (3%)
Ewing et al, 1976 (16)	2.75	1)Valsalva manouvre<br><br>2)BP response to sustained handgrip<br><br>3)Postural drop in blood pressure	NA	NA	NA	NA	1 or both AFT abnormal: 10/20 (50%)	Both AFT normal: 0/17 (0%)

Jermendy et al, 1991 (33)	5	1)Valsalva ratio (Parasympathetic)<br><br>2)30:15 ratio (Parasympathetic)<br><br>3)Postural drop in blood pressure (sympathetic)<br><br>4)Beat to beat variation (Parasympathetic)	Defined as<br><br>Normal: 0 score for parasympathetic tests<br><br>Borderline: 1 score for parasympathetic tests<br><br>Abnormal: 2 score for parasympathetic tests<br><br>*The patients were than assigned an autonomic function score from 0 to 6 and 3 groups were formed as patients without CAN (total score 0-1), patients with early (total score 2-3) and definitive (total score 4-6) signs of CAN<br><br>*Classification of patients was carried out on the basis of parasympathetic	NA	NA	NA	Early CAN: 2/13 (15%)<br><br>Definit e CAN: 10/17 (59%)	1/23 (4%)

			functional tests because no sympathetic function proved to be abnormal.					

* AFT, autonomic function test; CAN, cardiac autonomic neuropathy; CAN+, cardiac autonomic neuropathy positive; CAN-, cardiac autonomic neuropathy negative; CVE, cardiovascular disease events; CRR, cardiorespiratory reflex; HRV, heart rate variation; NA, information not available; QTc, corrected QT interval; SD, standard deviation.

Chowdhury M, et al. BMJ Open Diab Res Care 2021; 9:e002480. doi: 10.1136/bmjdrc-2021-002480

ESM Table 5 Participant metabolic characteristic from all final selected articles

Study	Population HbA1c (IFC mmol/mol or DCCT %)	Population fasting glucose (default is mmol/L, apart from where stated mg/dL)	Population eGFR (ml/min/1.73 m2)	Population creatinine clearance (mL/min, apart from where stated mL/s or mmol/L)	Population albuminuria (mg/mmol, apart from where stated mg/24h, g/24h or mg/day)	Population lipids (mg/dL or mmol/L)
Ko et al, 2008 (25)	9.0+/-2.2%	NA	NA	NA	NA	NA
Pop-Busui et al, 2017 (15)	CAN-: 66.0+/-17.0 mmol/mol (8.2+/-1.6 %)<br><br>CAN+: 74.0 +/- 20.0 mmol/mol (8.9 +/- 1.8 %)	NA	NA	NA	Sustained microalbuminuria (Sustained AER 30 mg/24h or more at two consecutive visits or ESRD defined as dialysis or renal transplantation):<br>CAN-: n=114<br><br>CAN+: n=35<br><br>Macroalbuminuria (AER 300 mg/24h or more, or ESRD):<br>Control: n=18<br><br>CAN: n=11	CAN-: Triglycerides 84.0+/-47.0 mg/dL, total cholesterol 181.0+/-33.0 mg/dL<br><br>CAN+: Triglycerides 100.0+/-58.0 mg/dL, total cholesterol 190.0+/-42.0 mg/dL

O'Brien et al, 1991 (22)	NA	NA	NA	NA	NA	CAN- cholesterol = 5.2 mmol/L<br><br>CAN+ cholesterol = 5.6 mmol/L
Cha et al, 2016 (31)	Whole cohort: 73.9+/-23.0 mmol/mol	Whole cohort: 9.87+/-4.51	Whole cohort: 83.1+/-18.0	NA	Whole cohort: 13.0 (7.0–54.9) mg/day	Whole cohort: HDL 1.07+/-0.30 mmol/L, LDL 2.85+/-0.85 mmol/L
Astrup et al, 2006 (24)	Diabetic nephropathy: 9.5 %+/-1.5<br><br>No diabetic nephropathy: 8.5%+/-1.1	NA	Diabetic nephropathy: 74.0+/-34.0<br><br>No diabetic nephropathy: NA	NA	Diabetic nephropathy: 796.0 (16–14,565) mg/24hr<br><br>No diabetic nephropathy: 8.0 (1–30) mg/24hr	Diabetic nephropathy: HDL 1.46+/-0.5, LDL 3.54+/-1.1 mmol/L, Triglycerides 1.22 (0.3–9.8) mmol/L<br><br>No diabetic nephropathy: HDL 1.56 +/- 0.4, LDL 2.82 +/- 0.9 mmol/L, Triglycerides 0.77 (0.28–3.1) mmol/L

Lykke et al, 2008 (26)	Normoalbuminuric group: 8.49+/-1.09%<br><br>Nephropathy group: 9.56+/-1.5%	NA	NA	NA	Normoalbuminuric group: Urinary albumin excretion 10.2 (range 1-40) mg/24h<br><br>Nephropathy: Urinary albumin excretion 1,609 (16-14545) mg/24h	Normoalbuminuric group: Cholesterol 4.76+/-0.98 mmol/L, HDL 1.56+/-0.51 mmol/L, Triglycerides 0.83 (range 0.28-3.05) mmol/L<br><br>Nephropathy group: Cholesterol: 5.64+/-1.22, HDL: 1.46+/-0.54 mmol/L, Triglycerides 1.41 (range 0.31–9.87) mmol/L
Okada et al, 2010 (29)	Preserved BRS = 8.1+/-1.8%<br><br>Depressed BRS = 8.3+/-1.9%	Preserved BRS: 147.0+/-45.0 mg/dL Depressed BRS: 159.0+/-53.0 mg/dL	NA	NA	NA	NA
Ziegler et al, 2008 (17)	NA	NA	NA	NA	NA	Whole cohort: total cholesterol 254.2+/-55.0 mg/dL, LDL: 161.5+/-45.1, HDL 49.3+/-15.4

Sawicki et al, 1996 (36)	Whole cohort: 8.9+/-2.0%	NA	NA	Whole cohort:1.08 (range 0.73-1.36) mL/s *median	NA	Whole cohort: 6.9+/-1.7 mmol/L
Young et al, 2009 (27)	No screening group (n=562): 7.0+/-1.5%<br><br>Screening (n=561): 7.2+/-1.6%	NA	NA	NA	NA	No screening (n=562): Triglycerides 168.0+/-101.0 mg/dL<br><br>Screening (n=561): Triglycerides 172.0+/-118.0 mg/dL
Sawicki et al, 1998 (37)	Whole cohort: 8.9% (8.1–10.5) *median (interquartile range)	NA	NA	NA	NA	Whole cohort: total cholesterol 6.1 (interquartile range 5.2–6.9) mmol/L
Rathmann et al, 1993 (35)	CAN-: 10.5+/-1.4%<br><br>CAN+: 11.6+/-1.0%	NA	NA	CAN-: 59.2 +/- 9.7<br><br>CAN+: 68.7 +/- 8.8	CAN- proteinuria: 0.06g/24h<br><br>CAN+ proteinuria: 0.15g/24h	CAN- triglycerides: 1.56+/-0.59 mmol/L, cholesterol 5.60+/-1.22 mmol/L<br><br>CAN+ triglycerides: 2.41+/-1.35 mmol/L, cholesterol 6.11 +/- 1.27 mmol/L

Jermendy et al, 1991 (33)	Total diabetic population: 7.1+/-0.2%	NA	NA	Total diabetic population: 87.0+/-4.0 mmol/L	NA	NA

* AER, albumin excretion rate; BRS, baroreflex sensitivity; CAN, cardiac autonomic neuropathy; CAN+, cardiac autonomic neuropathy positive; CAN-, cardiac autonomic neuropathy negative; eGFR, estimated glomerular filtration rate; ESRD, end stage renal disease; HDL, high density lipoprotein; LDL, low density lipoprotein; NA, information not available

* Studies without reported metabolic characteristics: Chen et al, 2001 (32), Vujosevic et al, 2012 (30), Valensi et al, 2001 (20), Soedamah-Muthu et al, 2008 (13), Lee et al, 2003 (19), Ewing et al, 1980 (18), Cohen et al, 2003 (23), Navarro et al, 1996 (34), Sampson et al, 1990 (21), Töyry et al. 1996 (14), Veglio et al, 2000 (38), Pop-Busui et al, 2010 (28), Ewing et al, 1976 (16)

ESM Table 6 Summary of the overall risk of bias assessment

Study	Overall risk of bias
Chen et al, 2001 (33)	Moderate
Ko et al, 2008 (26)	Moderate
Pop-Busui et al, 2017 (16)	Low
Vujosevic et al, 2012 (31)	Moderate
Valensi et al, 2001 (21)	Moderate
Soedamah-Muthu et al, 2008 (14)	Moderate
O'Brien et al, 1991 (23)	Serious
Lee et al, 2003 (20)	Moderate
Ewing et al, 1980 (19)	Low
Cha et al, 2016 (32)	Moderate
Astrup et al, 2006 (25)	Moderate
Lykke et al, 2008 (27)	Moderate
Cohen et al, 2003 (24)	Moderate
Okada et al, 2010 (30)	Moderate
Navarro et al, 1996 (35)	Serious
Sampson et al, 1990 (22)	Moderate
Töyry et al. 1996 (15)	Moderate

Chowdhury M, *et al. BMJ Open Diab Res Care* 2021; 9:e002480. doi: 10.1136/bmjdrc-2021-002480

Veglio et al, 2000 (39)	Moderate
Ziegler et al, 2008 (18)	Moderate
Sawicki et al, 1996 (37)	Moderate
Young et al, 2009 (28)	Moderate
Sawicki et al, 1998 (38)	Moderate
Pop-Busui et al, 2010 (29)	Moderate
Rathmann et al, 1993 (36)	Serious
Ewing et al, 1976 (17)	Moderate
Jermendy et al, 1991 (34)	Moderate

ESM Figure 1 Prisma flowchart demonstrating the article screening process

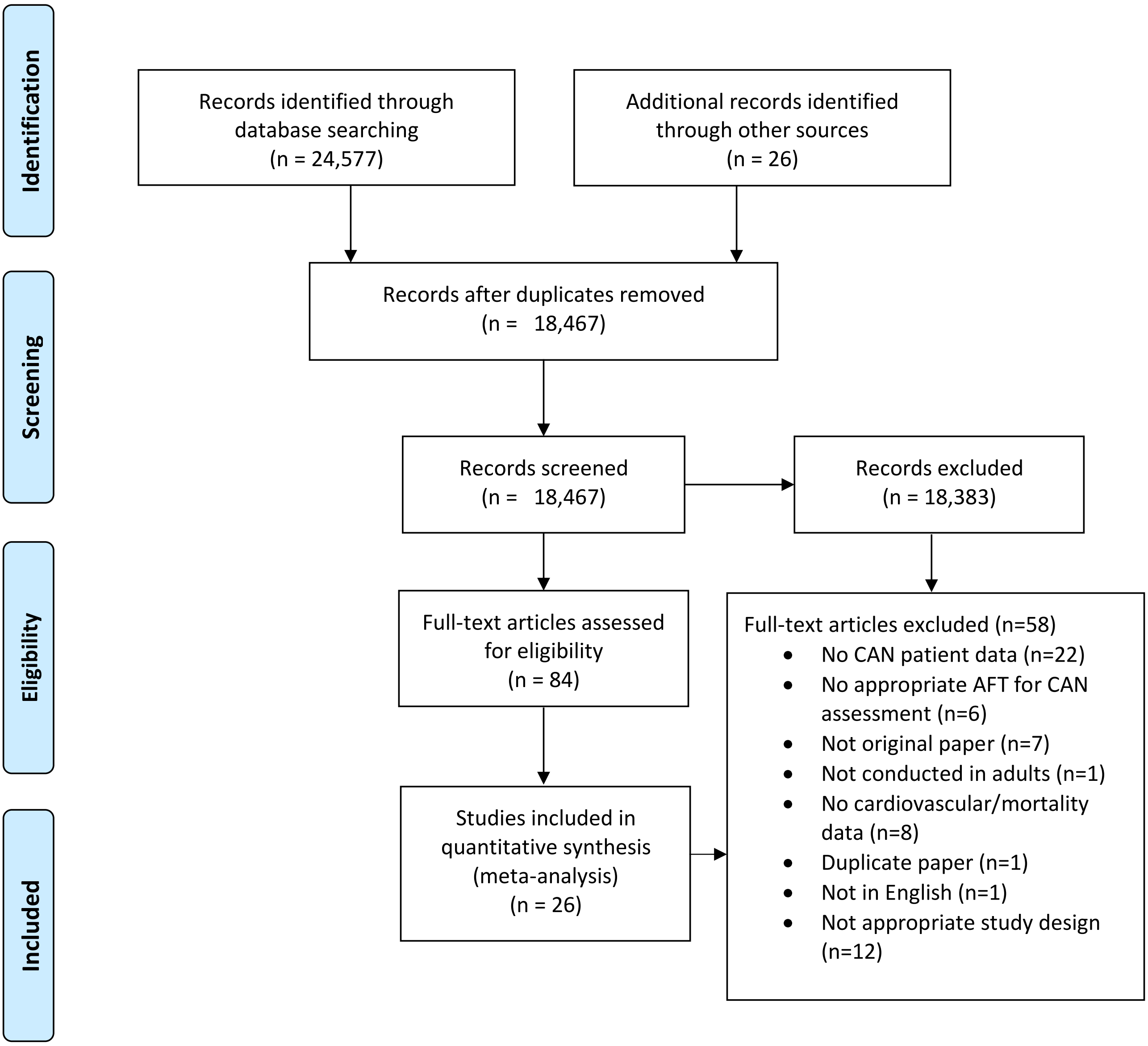

ESM Figure 2 A Forest plot including relative risk and 95% CI for subgroup analyses with cardiac autonomic neuropathy and cardiovascular disease events based on the aetiology of diabetes.

	CAN		Control			Risk Ratio	
**Study or Subgroup**	**Events**	**Total**	**Events**	**Total**	**Weight**	**M-H, Random, 95% CI**	**Year**
**1.1.1 Type 1 diabetes**							
Sampson et al 1990	4	73	0	38	0.8%	4.74 [0.26, 85.85]	1990
O'Brien et al 1991	8	84	7	422	5.1%	5.74 [2.14, 15.41]	1991
Astrup et al 2006	91	281	6	107	6.9%	5.78 [2.61, 12.80]	2006
Lykke et al 2008	14	34	1	100	1.6%	41.18 [5.62, 301.56]	2008
Pop-Busui et al 2017	49	131	191	1262	15.8%	2.47 [1.91, 3.20]	2017
**Subtotal (95% CI)**		**603**		**1929**	**30.2%**	**5.54 [2.28, 13.45]**	
Total events	166		205				
Heterogeneity: Tau^2 = 0.63; Chi^2 = 16.77, df = 4 (P = 0.002); I^2 = 76%							
Test for overall effect: Z = 3.78 (P = 0.0002)							
**1.1.2 Type 2 diabetes**							
Töyry et al 1996	6	19	5	77	4.5%	4.86 [1.66, 14.25]	1996
Lee et al 2003	13	78	0	68	0.8%	23.58 [1.43, 389.41]	2003
Cohen et al 2003	26	405	14	467	8.9%	2.14 [1.13, 4.04]	2003
Ko et al 2008	97	627	34	499	13.6%	2.27 [1.56, 3.30]	2008
Young et al 2009	17	245	15	878	8.3%	4.06 [2.06, 8.01]	2009
Pop-Busui et al 2010	23	572	140	7563	12.4%	2.17 [1.41, 3.35]	2010
Okada et al 2010	15	85	4	99	4.6%	4.37 [1.51, 12.66]	2010
Vujosevic et al 2012	16	51	4	25	5.1%	1.96 [0.73, 5.25]	2012
Cha et al 2016	65	117	13	42	11.5%	1.79 [1.11, 2.90]	2016
**Subtotal (95% CI)**		**2199**		**9718**	**69.8%**	**2.45 [1.93, 3.11]**	
Total events	278		229				
Heterogeneity: Tau^2 = 0.02; Chi^2 = 9.81, df = 8 (P = 0.28); I^2 = 18%							
Test for overall effect: Z = 7.36 (P < 0.00001)							
**Total (95% CI)**		**2802**		**11647**	**100.0%**	**2.96 [2.27, 3.84]**	
Total events	444		434				
Heterogeneity: Tau^2 = 0.10; Chi^2 = 25.69, df = 13 (P = 0.02); I^2 = 49%							
Test for overall effect: Z = 8.09 (P < 0.00001)							
Test for subgroup differences: Chi^2 = 3.03, df = 1 (P = 0.08), I^2 = 67.0%							

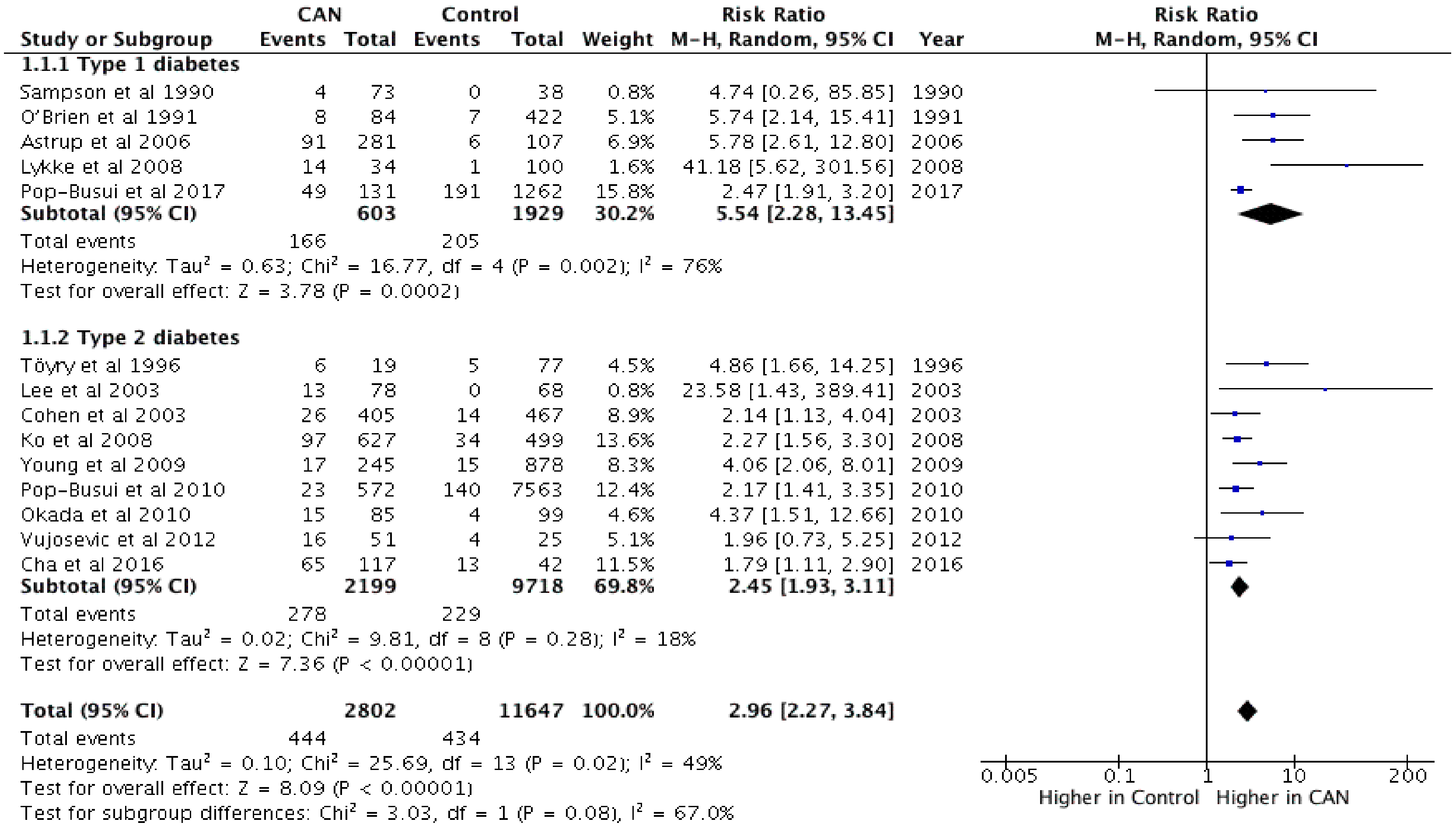

ESM Figure 3 A Forest plot including relative risk and 95% CI for subgroup analyses of studies with cardiac autonomic neuropathy and future cardiovascular disease events based on number of autonomic function test abnormalities (1 vs ≥2 abnormalities).

	CAN		Control			Risk Ratio	
**Study or Subgroup**	**Events**	**Total**	**Events**	**Total**	**Weight**	**M-H, Random, 95% CI**	**Year**
**1.1.1 Minimum 1 positive AFT**							
Sampson et al 1990	4	73	0	38	0.8%	4.74 [0.26, 85.85]	1990
Töyry et al 1996	6	19	5	77	4.3%	4.86 [1.66, 14.25]	1996
Cohen et al 2003	26	405	14	467	8.5%	2.14 [1.13, 4.04]	2003
Astrup et al 2006	91	281	6	107	6.6%	5.78 [2.61, 12.80]	2006
Ko et al 2008	97	627	34	499	13.0%	2.27 [1.56, 3.30]	2008
Young et al 2009	17	245	15	878	8.0%	4.06 [2.06, 8.01]	2009
Okada et al 2010	15	85	4	99	4.4%	4.37 [1.51, 12.66]	2010
Cha et al 2016	22	48	13	42	9.9%	1.48 [0.86, 2.56]	2016
**Subtotal (95% CI)**		**1783**		**2207**	**55.5%**	**2.88 [2.01, 4.12]**	
Total events	278		91				
Heterogeneity: Tau² = 0.11; Chi² = 13.19, df = 7 (P = 0.07); I² = 47%							
Test for overall effect: Z = 5.79 (P < 0.00001)							
**1.1.2 Minimum 2 positive AFT**							
O'Brien et al 1991	8	84	7	422	4.9%	5.74 [2.14, 15.41]	1991
Lykke et al 2008	14	34	1	100	1.5%	41.18 [5.62, 301.56]	2008
Pop-Busui et al 2010	23	572	140	7563	11.9%	2.17 [1.41, 3.35]	2010
Cha et al 2016	43	69	13	42	10.9%	2.01 [1.24, 3.28]	2016
Pop-Busui et al 2017	49	131	191	1262	15.2%	2.47 [1.91, 3.20]	2017
**Subtotal (95% CI)**		**890**		**9389**	**44.5%**	**2.84 [1.84, 4.38]**	
Total events	137		352				
Heterogeneity: Tau² = 0.14; Chi² = 12.01, df = 4 (P = 0.02); I² = 67%							
Test for overall effect: Z = 4.72 (P < 0.00001)							
**Total (95% CI)**		**2673**		**11596**	**100.0%**	**2.81 [2.17, 3.63]**	
Total events	415		443				
Heterogeneity: Tau² = 0.10; Chi² = 25.43, df = 12 (P = 0.01); I² = 53%							
Test for overall effect: Z = 7.88 (P < 0.00001)							
Test for subgroup differences: Chi² = 0.00, df = 1 (P = 0.96), I² = 0%							

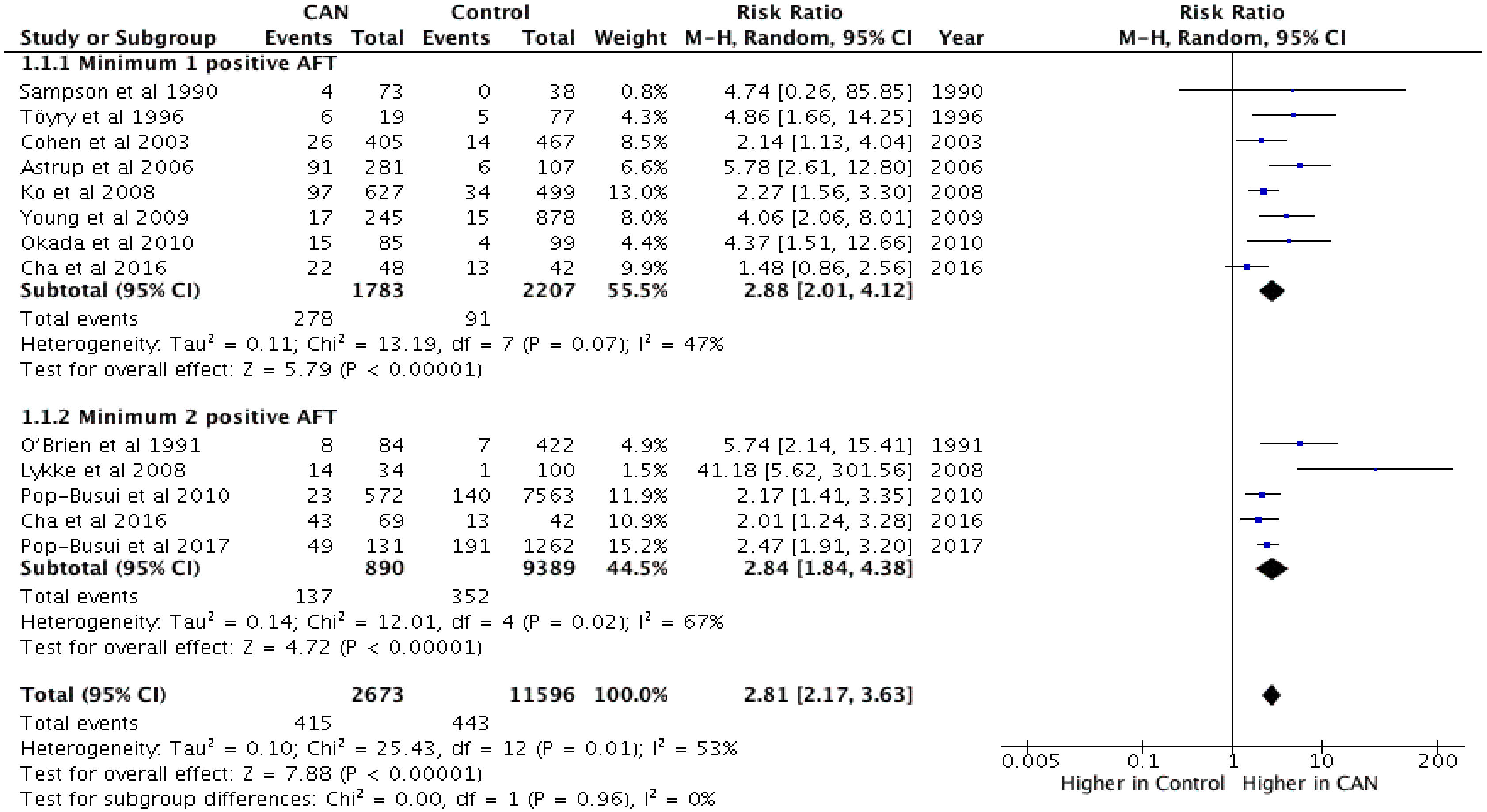

Chowdhury M, *et al. BMJ Open Diab Res Care* 2021; 9:e002480. doi: 10.1136/bmjdrc-2021-002480

ESM Figure 4 A Forest plot including relative risk and 95% CI for subgroup analyses of studies with cardiac autonomic neuropathy and all-cause mortality based on the aetiology of diabetes.

	CAN		Control			Risk Ratio
**Study or Subgroup**	**Events**	**Total**	**Events**	**Total**	**Weight**	**M-H, Random, 95% CI**
**1.3.1 Type 1 diabetes**						
Astrup et al 2006	69	281	6	107	7.0%	4.38 [1.96, 9.78]
Lykke et al 2008	59	291	3	100	5.8%	6.76 [2.17, 21.08]
Navarro et al 1996	110	417	6	128	7.0%	5.63 [2.54, 12.49]
O'Brien et al 1991	23	84	21	422	7.9%	5.50 [3.20, 9.47]
Pop-Busui et al 2017	4	131	13	1262	5.9%	2.96 [0.98, 8.96]
Sampson et al 1990	20	73	4	38	6.3%	2.60 [0.96, 7.07]
Sawicki et al 1996	16	26	17	59	8.0%	2.14 [1.29, 3.53]
Soedamah-Muthu et al 2008	68	941	34	1846	8.2%	3.92 [2.62, 5.88]
Veglio et al 2000	10	75	10	241	6.9%	3.21 [1.39, 7.42]
**Subtotal (95% CI)**		**2319**		**4203**	**62.9%**	**3.76 [2.89, 4.91]**
Total events	379		114			
Heterogeneity: $Tau^2 = 0.04$; $Chi^2 = 10.39$, df = 8 (P = 0.24); $I^2 = 23\%$						
Test for overall effect: Z = 9.78 (P < 0.00001)						
**1.3.2 Type 2 diabetes**						
Chen et al 2001	106	371	29	241	8.3%	2.37 [1.63, 3.46]
Lee et al 2003	15	78	2	68	4.8%	6.54 [1.55, 27.57]
Pop-Busui et al 2010	38	572	291	7563	8.4%	1.73 [1.24, 2.39]
Sawicki et al 1998	58	84	100	132	8.7%	0.91 [0.77, 1.08]
Vujosevic et al 2012	24	51	5	25	6.9%	2.35 [1.02, 5.43]
**Subtotal (95% CI)**		**1156**		**8029**	**37.1%**	**1.94 [1.03, 3.65]**
Total events	241		427			
Heterogeneity: $Tau^2 = 0.41$; $Chi^2 = 47.46$, df = 4 (P < 0.00001); $I^2 = 92\%$						
Test for overall effect: Z = 2.05 (P = 0.04)						
**Total (95% CI)**		**3475**		**12232**	**100.0%**	**3.02 [1.89, 4.83]**
Total events	620		541			
Heterogeneity: $Tau^2 = 0.65$; $Chi^2 = 148.55$, df = 13 (P < 0.00001); $I^2 = 91\%$						
Test for overall effect: Z = 4.61 (P < 0.00001)						
Test for subgroup differences: $Chi^2 = 3.60$, df = 1 (P = 0.06), $I^2 = 72.2\%$						

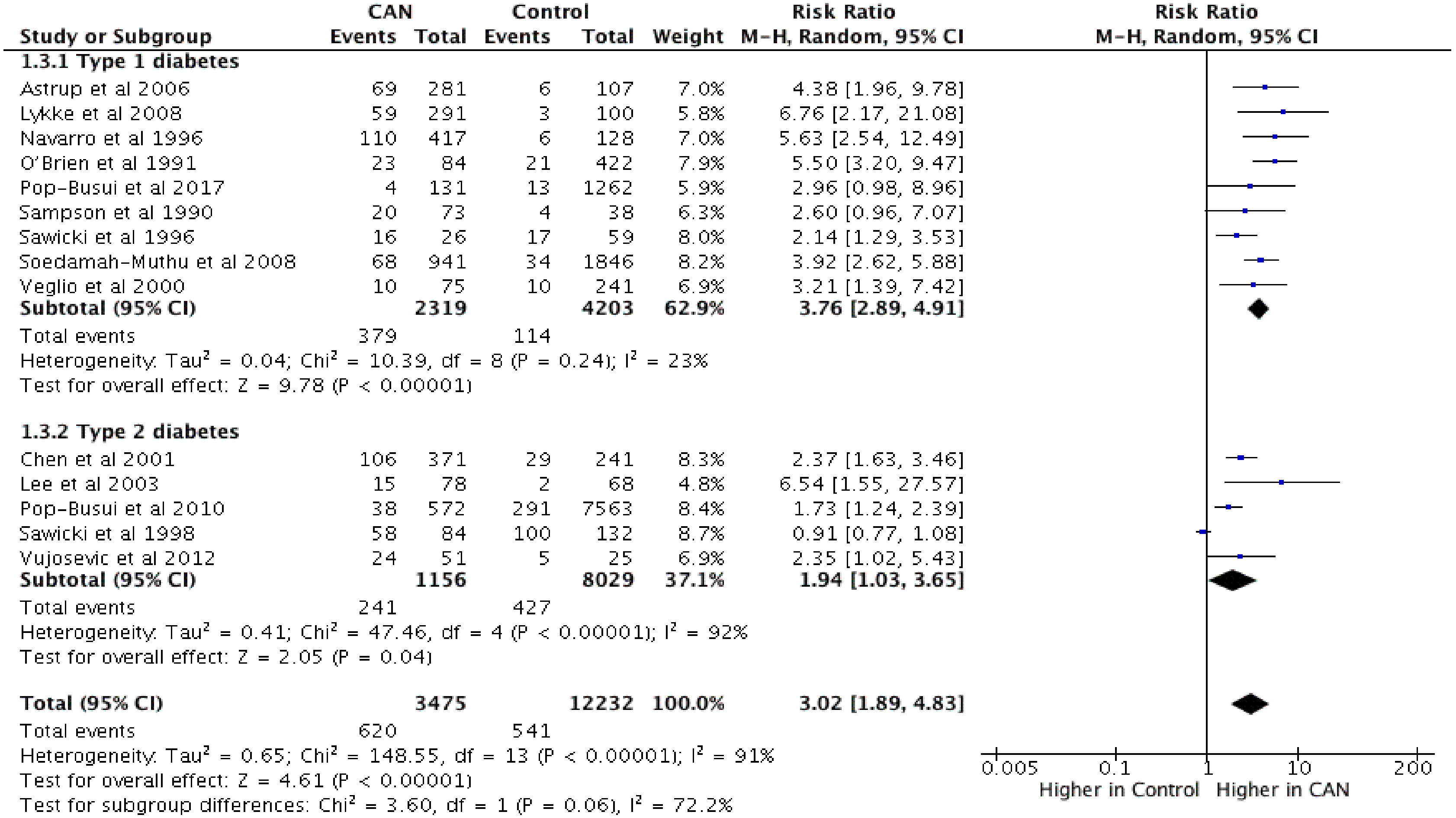

ESM Figure 5 A Forest plot including relative risk and 95% CI for sensitivity analysis of studies with cardiac autonomic neuropathy and future cardiovascular events after removing three studies at high risk of bias.

	CAN		Control			Risk Ratio	
**Study or Subgroup**	**Events**	**Total**	**Events**	**Total**	**Weight**	**M-H, Random, 95% CI**	**Year**
Ewing et al 1980	8	40	2	33	2.8%	3.30 [0.75, 14.49]	1980
Sampson et al 1990	4	73	0	38	0.8%	4.74 [0.26, 85.85]	1990
Töyry et al 1996	6	19	5	77	4.6%	4.86 [1.66, 14.25]	1996
Valensi et al 2001	22	33	3	42	4.4%	9.33 [3.06, 28.51]	2001
Cohen et al 2003	26	405	14	467	8.8%	2.14 [1.13, 4.04]	2003
Lee et al 2003	13	78	0	68	0.9%	23.58 [1.43, 389.41]	2003
Astrup et al 2006	91	281	6	107	6.9%	5.78 [2.61, 12.80]	2006
Ko et al 2008	97	627	34	499	12.9%	2.27 [1.56, 3.30]	2008
Lykke et al 2008	14	34	1	100	1.7%	41.18 [5.62, 301.56]	2008
Young et al 2009	17	245	15	878	8.2%	4.06 [2.06, 8.01]	2009
Pop-Busui et al 2010	23	572	140	7563	11.9%	2.17 [1.41, 3.35]	2010
Okada et al 2010	15	85	4	99	4.7%	4.37 [1.51, 12.66]	2010
Vujosevic et al 2012	16	51	4	25	5.2%	1.96 [0.73, 5.25]	2012
Cha et al 2016	65	117	13	42	11.1%	1.79 [1.11, 2.90]	2016
Pop-Busui et al 2017	49	131	191	1262	14.8%	2.47 [1.91, 3.20]	2017
**Total (95% CI)**		**2791**		**11300**	**100.0%**	**3.05 [2.32, 4.00]**	
Total events	466		432				

Heterogeneity: $Tau^2 = 0.11$; $Chi^2 = 28.90$, df = 14 (P = 0.01); $I^2 = 52\%$

Test for overall effect: Z = 8.06 (P < 0.00001)

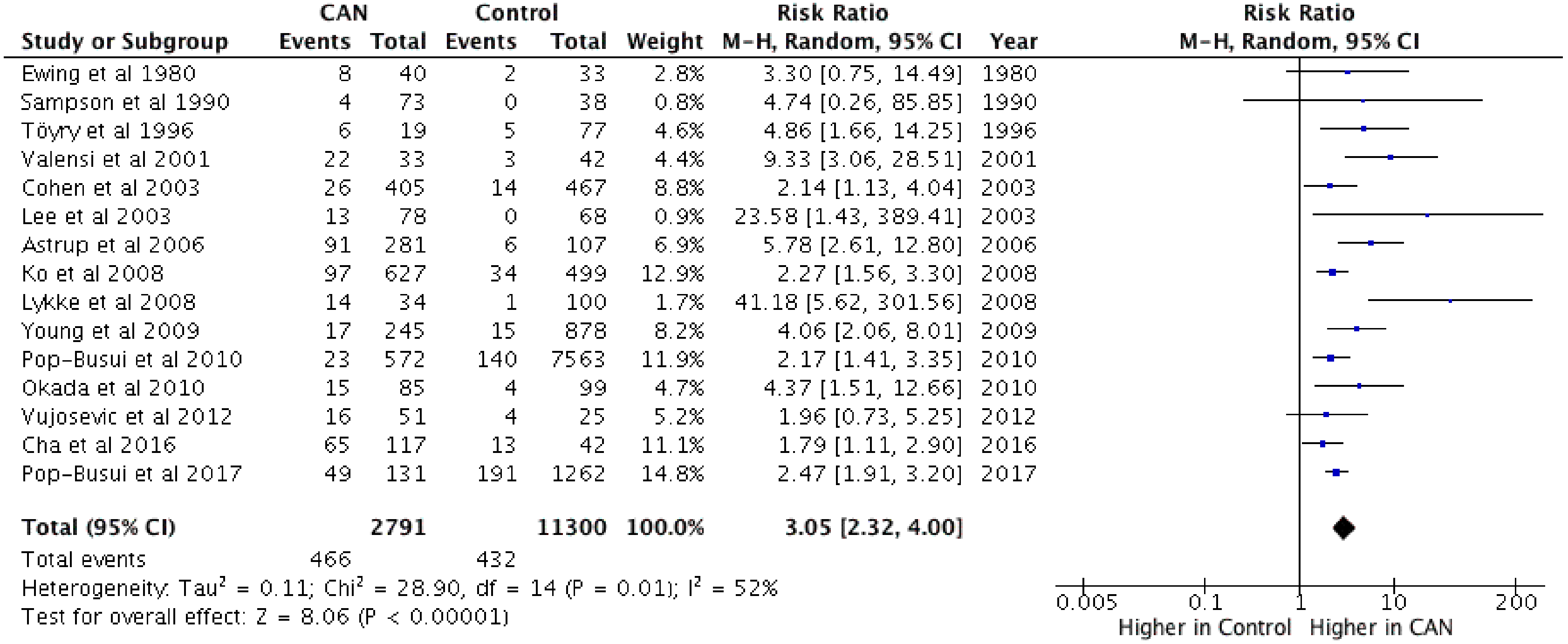

ESM Figure 6 A Forest plot including relative risk and 95% CI for sensitivity analysis of studies with cardiac autonomic neuropathy and all-cause mortality after removing three studies at high risk of bias.

	CAN		Control			Risk Ratio
**Study or Subgroup**	**Events**	**Total**	**Events**	**Total**	**Weight**	**M-H, Random, 95% CI**
Astrup et al 2006	69	281	6	107	6.6%	4.38 [1.96, 9.78]
Chen et al 2001	106	371	29	241	8.1%	2.37 [1.63, 3.46]
Ewing et al 1976	10	20	0	17	1.8%	18.00 [1.13, 286.20]
Ewing et al 1980	21	40	5	33	6.4%	3.46 [1.47, 8.18]
Jermendy et al 1991	12	30	1	23	3.0%	9.20 [1.29, 65.73]
Lee et al 2003	15	78	2	68	4.3%	6.54 [1.55, 27.57]
Lykke et al 2008	59	291	3	100	5.3%	6.76 [2.17, 21.08]
Pop-Busui et al 2010	38	572	291	7563	8.3%	1.73 [1.24, 2.39]
Pop-Busui et al 2017	4	131	13	1262	5.4%	2.96 [0.98, 8.96]
Sampson et al 1990	20	73	4	38	5.8%	2.60 [0.96, 7.07]
Sawicki et al 1996	16	26	17	59	7.7%	2.14 [1.29, 3.53]
Sawicki et al 1998	58	84	100	132	8.6%	0.91 [0.77, 1.08]
Soedamah-Muthu et al 2008	68	941	34	1846	8.1%	3.92 [2.62, 5.88]
Veglio et al 2000	10	75	10	241	6.5%	3.21 [1.39, 7.42]
Vujosevic et al 2012	24	51	5	25	6.5%	2.35 [1.02, 5.43]
Ziegler et al 2008	30	79	19	80	7.8%	1.60 [0.99, 2.59]
**Total (95% CI)**		**3143**		**11835**	**100.0%**	**2.81 [1.85, 4.27]**
Total events	560		539			

Heterogeneity: $Tau^2 = 0.52$; $Chi^2 = 125.74$, df = 15 ($P < 0.00001$); $I^2 = 88\%$
Test for overall effect: $Z = 4.85$ ($P < 0.00001$)

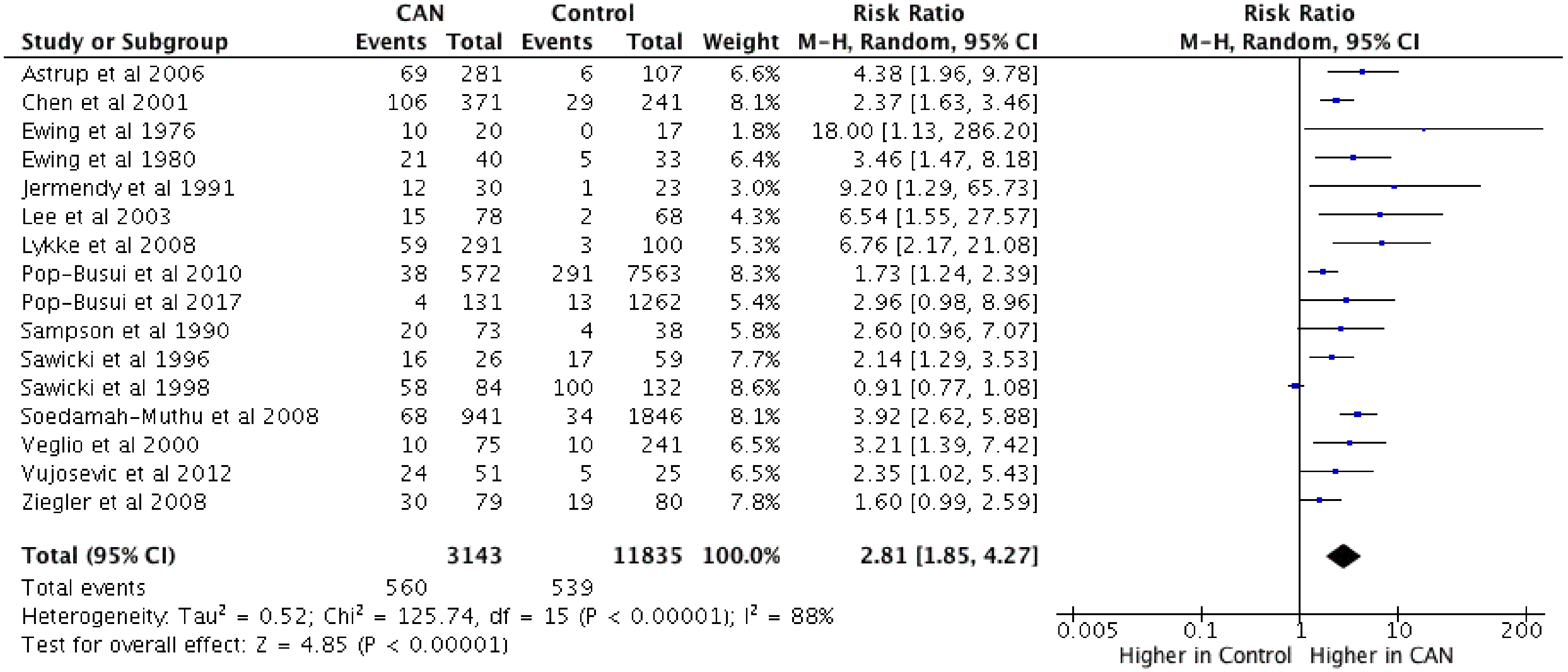

Supplement: Supplementary data [file bmjdrc-2021-002480supp001.pdf]
